# β-Glucosidase and Its Application in Bioconversion of Ginsenosides in *Panax ginseng*

**DOI:** 10.3390/bioengineering10040484

**Published:** 2023-04-18

**Authors:** Thi Ngoc Anh Tran, Jin-Sung Son, Muhammad Awais, Jae-Heung Ko, Deok Chun Yang, Seok-Kyu Jung

**Affiliations:** 1 Graduate School of Biotechnology, College of Life Science, Kyung Hee University, Yongin 17104, Republic of Korea; 2 Department of Plant & Environmental New Resources, Kyung Hee University, Yongin 17104, Republic of Korea; 3 Department of Horticulture, Kongju National University, Yesan 32439, Republic of Korea

**Keywords:** β-glucosidase, *Panax ginseng*, ginsenosides, bioconversion, recombinant enzyme

## Abstract

Ginsenosides are a group of bioactive compounds isolated from *Panax ginseng*. Conventional major ginsenosides have a long history of use in traditional medicine for both illness prevention and therapy. Bioconversion processes have the potential to create new and valuable products in pharmaceutical and biological activities, making them both critical for research and highly economic to implement. This has led to an increase in the number of studies that use major ginsenosides as a precursor to generate minor ones using β-glucosidase. Minor ginsenosides may also have useful properties but are difficult to isolate from raw ginseng because of their scarcity. Bioconversion processes have the potential to create novel minor ginsenosides from the more abundant major ginsenoside precursors in a cost-effective manner. While numerous bioconversion techniques have been developed, an increasing number of studies have reported that β-glucosidase can effectively and specifically generate minor ginsenosides. This paper summarizes the probable bioconversion mechanisms of two protopanaxadiol (PPD) and protopanaxatriol (PPT) types. Other high-efficiency and high-value bioconversion processes using complete proteins isolated from bacterial biomass or recombinant enzymes are also discussed in this article. This paper also discusses the various conversion and analysis methods and their potential applications. Overall, this paper offers theoretical and technical foundations for future studies that will be both scientifically and economically significant.

## 1. Introduction

### Ginseng and Ginsenosides

In the *Panax* genus, 13 Panax species were identified (accessed on 17 April 2023 from http://www.theplantlist.org). Some *Panax* species have a long history of use in traditional medicine due to their composition and functionality [1]. Korean ginseng (*Panax ginseng*) is considered one of the most precious medicinal plants, owing to the abundance of ginsenosides of highly economical and medicinal value; however, this form of ginseng also contains additional potentially useful secondary compounds, including polysaccharides, proteins, peptides, amino acids, polyphenols, polyacetylenes, alkaloids, lipids, essential oils, phytosterols, organic acids, and terpenes [2,3,4,5,6,7]. Many such compounds, including caffeic acid, vanillic acid, coumaric acid, ferulic acid, gentisic acid, cinnamic acid, and polyactetylenes, have been reported to possess biological and pharmacological activity as antioxidants, anti-inflammatory, and/or anticancer agents [2,4,8,9,10]. *P. ginseng* has been utilized to investigate various pharmaceutical applications, including use as antioxidants, anticancer, anti-inflammatory, anti-hyperglycemic, treating cardiovascular and cerebrovascular diseases, and boosting the immune system [1,2,4,5].

Ginsenosides are recognized as the main bioactive components; after extraction and purification, the concentrations of total and individual ginsenosides serve as benchmarks to determine the quality of the final product. Based on the structure and derivatives, ginsenosides can be divided into three main groups: protopanaxadiol (PPD)-type, protopanaxatriol (PPT)-type, and oleanane-type. The PPD-type and PPT-type ginsenosides are classified based on the sugar moieties that are attached at the C-3, C-6, and/or C-20 positions while oleanolic acid is derived from β-amyrin [2,4,11]. In addition, the spatial distribution of ginsenosides in various parts of the ginseng plant has been a subject of some debate, with the explanation perhaps being a physiological function during tissue formation and development [12]. Furthermore, ginsenosides differ between species, and even within the same species, ginseng grown under different geographical and meteorological conditions can produce secondary compounds with variable chemical compositions and quantities [2,4,6,11,12,13].

**Figure 1:**
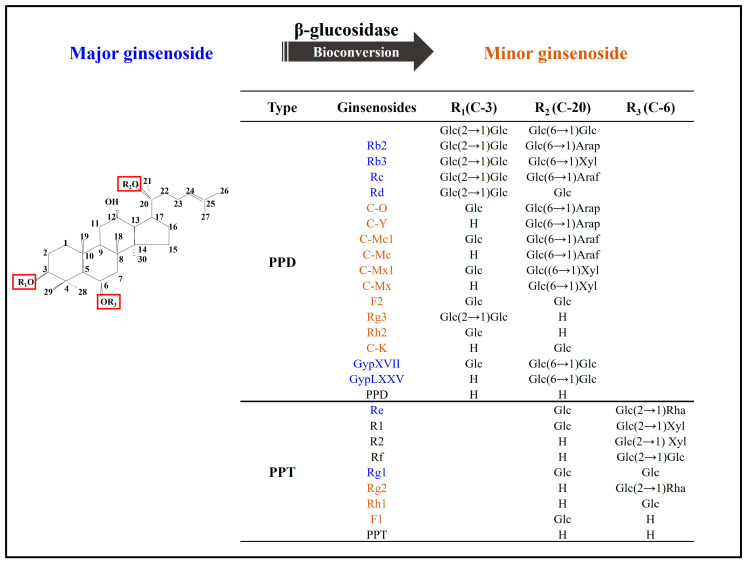
Different types of ginsenosides that may be candidates for bioconversion. Ginsenosides are discriminated by the type, number of sugar moieties, and the attracted position. The major and minor ginsenosides are marked in blue and orange, respectively. The abbreviation of sugar moieties are Glc: D-glucose, Arap: L-arabinopyranoside, Araf: L-arabinofuranoside; Xyl: D-xylose; Rha: L-rhamnose.

In recent decades, the application of biotechnological techniques for ginsenoside production has become commonplace, including tissue culture to prepare biomass and stable materials for secondary compound collection, chemical elicitor treatment to increase ginsenoside synthesis in cultured ginseng cells, or adventitious roots through activation of phenylalanine ammonia lyases or the signal transducer nitric oxide. Transgenic plants are regenerated by overexpression of ginsenoside synthesis genes in plant cells, which is another efficient biotechnological way for enhancing ginsenoside yield [8]. In terms of enhancing ginsenosides yield and activities, the bioconversion of ginsenosides using microbial or recombinant enzymes is an effective method to convert major ginsenosides to minor ginsenosides that are normally present at low concentrations in fresh ginseng (Figure 1) [2]. The major ginsenosides identified to date are Rb1, Rb2, Rb3, Rc, Rd, Re, and Rg1. Through the bioconversion, the large changes are the elimination of sugar moiety at C-20, and subsequently at C-6 or C-3 of these component indicated above. Consequently, minor ginsenosides are generated and have deglycosylated forms, including Rg3, Rh2, Rh1, F2, Rg2, Rg5, F1 compound (C)-Mc1, C-Mc, C-O, C-Y, and C-K (Figure 1) [5,14,15]. The use of bioconversion to produce minor ginsenosides may help increase the pharmaceutical applications of such compounds and scientific research in this field [2,4,8,9,16].

New approaches are being developed to isolate, purify, and quantify ginsenosides on the basis of their structures and properties. The origin and type of ginseng explants have a direct impact on the extraction technique and its effectiveness. Using the polar characteristics of the solvent is the most common technique used to extract total ginsenosides. In addition to the concentration and type of solvent, the solvent/material ratio and extraction duration may influence the results of extraction and purification. Besides, several physical technologies, such as reflux systems, sonication, and rotary evaporator systems, are used independently or in combination to extract ginsenosides and optimize the resulting content [17,18,19]. For purification, many specific methods are used to increase the recovery and purity of single ginsenosides. For example, linear gradient counter-current chromatography effectively separates ginsenoside compounds extracted from the roots of *Panax quinquefolium* with a broad range of partition coefficients, including 11 main ginsenosides (ginsenosides Rg and Re; acetyl ginsenosides Rg1, Rb1, Rc, Rg2, and Rb3; quinquefoliums R1 and Rd; gypenoside XVII; notoginsenoside Fd) [20]. In this same species of ginseng, the use of high-performance centrifugal partition chromatography (HPCPC) combined with evaporative light-scattering detection (ELSD) was developed for the separation and purification of three compounds (Rc, Rb1, and Re) [21]. For another species of ginseng, ginsenoside F5 and F3 isomeric compounds were initially isolated and purified by reverse-phase high-performance liquid chromatography (RP-HPLC) from crude extracts of flower buds of *Panax ginseng*; later, the process was optimized employing a ZORBAX Eclipse XDB C-18 column and DAISOGEL C-18 column using various gradient elution systems and flow rates [22].

Bioconversion processes for ginsenosides can be evaluated quantitatively and qualitatively. Several analytical techniques can be used, such as thin layer chromatography (TLC), high performance thin layer chromatography (HPTLC), high-speed counter current chromatography (HSCCC), high performance centrifugal partition chromatography (HPCPC), gas chromatography (GC), high performance liquid chromatography (HPLC), ultra-performance liquid chromatography (UPLC), liquid chromatography mas spectrometry/mass spectrometry (LC-MS/MS), nuclear magnetic resonance (NMR), and two-dimensional nuclear magnetic resonance (2D-NMR). These approaches can be used separately or in combination to characterize complex natural compounds with great sensitivity, specificity, and adaptability [4,14]. The results of these analyses may contribute to future molecular biology, genomics, transcriptomics, proteomics, and metabolomics studies [12,13].

## 2. β-Glucosidases and Their Functions

β-glucosidase (EC 3.2.1.21) enzymes belong to the large glycoside hydrolase (GH) family of enzymes that hydrolyze the glycosidic bond. Glycoside hydrolases are classified based on amino acid sequence similarity and conserved motifs. Under this system, 180 GH families are listed in the Carbohydrate Active enZYme (CAZY) Website (http://www.cazy.org/, accessed on 14 April 2023) [9,10,12,23]. The β-glucosidases have been categorized into the following families: GH1, GH2, GH3, GH5, GH9, GH30, GH39, and GH116. The hydrolysis of terminal and non-reducing β-D-glycosyl residues by β-glucosidase releases β-D-glucose from glycosides and/or oligosaccharides. The β-glucosidase is found in all domains of living organisms and possesses several functions such as glycolipid breakdown and exogenous glycoside metabolism in animals. In plants, the function of β-glucosidase has been reported to involve processes such as cell wall lignification, cell wall β-glucan turnover, phytohormone activation, secondary metabolism, and aromatic compound release. In addition, it participates in both sides of plant–microbe and plant–insect interactions. Furthermore, biomass conversion was also described as a function of β-glucoside in microorganisms [10,23,24]. As a result, β-glucosidase can be obtained from the crude protein of most living organisms: microorganisms are the preferred source due to their high biomass potential. Moreover, to obtain significant yields of protein with high purity, protein biosynthetic genes were cloned into the target vector and placed under the control of a strong promoter before being transferred to a host with high protein production and biomass yield. The next step is to lyse the host cell and separate the target protein from other proteins depending on the structure and components of the vector carrying the gene, such as a specific binding domain or tag [15,24,25,26,27,28,29]. 

The application of β-glucosidase in living organisms is also garnering more interest. The β-glucosidase used in biomass conversion is often isolated from bacteria or fungi [2,6,9,10]. Isolated β-glucosidase is a valuable enzyme for the food, waste, and biofuel processing industry [2,30,31]. For example, the application of β-glucosidase activity from microorganisms isolated from food sources is receiving increasing attention due to its safe and useful features. To illustrate, β-glucosidase obtained from *Lactobacillus rhamnosus* isolated from fermented soymilk has great potential to enrich bioactive isoflavones for the development of functional fermented soy-based products [32].

The hydrolysis processes mediated by β-glucosidase have been used to modify flavor precursors to enhance the quality of food and beverage. Moreover, proteins with desirable properties may be targeted to increase their abundance in plants or for overproduction in transgenic microbial or plant hosts, as well as for engineering to improve their catalytic properties for flavor enhancement, stability, nutritional improvement, and to aid in plant disease prevention [2,10,33,34]. As β-glucosidase exhibits multiple activities and functions in microorganisms, plants, animals, and humans, it is anticipated to be used in many additional, as well as continued, current applications [6,10,33].

## 3. Bioconversion of Ginsenosides

In recent decades, numerous studies have confirmed the successful chemical, physical, and biological transformation of major ginsenosides into minor ginsenosides, resulting in increased pharmacological activity (Figure 1) [2,4,8,35]. The biologically significant transformation method utilizes a biological process that has its own unique significance in maintaining its essential activities. Biotransformation involves enzymatic hydrolysis among intestinal bacteria for selective conversion to mimic biological conditions of endophytic bacteria, edible bacteria, soil microbes, etc., and could prove useful in multiple industrial sectors. Enzymatic conversions are considered safe and commercially viable biotransformation mechanisms whose products are safe for human consumption and use [2,4,9]. Microbial transformation and recombinant enzymes are effective methods to induce high-quality for bioconversion and are undergoing extensive study, particularly with regard to post-conversion specificity of substrates and products [2,4,5,9,10].

In the ginseng industry, PPD-type and PPT-type ginsenosides from *P. ginseng* are successfully converted to minor ginsenosides using microbes and enzymatic methods. In ginsenosides, the sugar moiety consists of 1∼4 molecules of glycosides and common sugars are D-glucose, L-arabinopyranoside, L-arabinofuranoside, D-xylose, and/or L-rhamnose. Several enzymes, including β-glucosidase, β-xylosidase, α-L-arabinofuranosidase, and α-L-rhamnosidase, have the ability to convert ginsenoside compounds based on their characteristic sugar moieties [5,36]. Intriguingly, β-glucosidase is the most prevalent enzyme with a significant and crucial role in the generation of valuable compounds that are otherwise available in only limited quantities within extracted materials [2,10,29,32,37,38,39,40,41,42,43,44,45,46]. The mechanism of β-glucosidase can be classified as two reactions: glycoside hydrolase (GH) and glycoside transferase (GT) activities; however, GT activities and its application are not commonly exploited due to its sophisticated synthesis and the high cost of the reaction [36,47,48].

**Figure 2:**
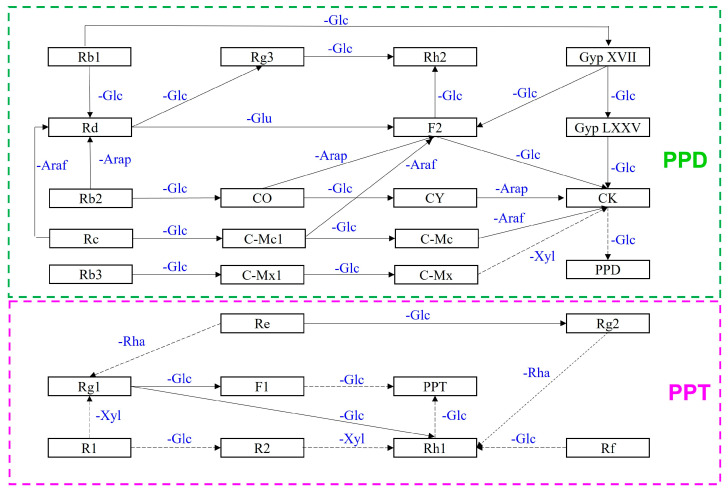
Overview of ginsenoside bioconversion. The PPD and PPT pathways are indicated in the green and pink boxes, respectively. The changed sugar moieties are showed in blue. The abbreviations of sugar moieties are as follows: Glc: D-glucose, Arap: L-arabinopyranoside, Araf: L-arabinofuranoside; Xyl: D-xylose; Rha: L-rhamnose. The marked ‘’-‘’ before sugar moieties indicted the hydrolysis. The lid arrows represent confirmed conversion pathways. Interrupted arrows indicate predicted biotransformation pathways.

Since Rb1 is the most abundant compound in ginseng, it is frequently employed as the primary substrate and initiator in bioconversion reactions. The Rb1 molecule consists of two glucose molecules at the C3 position and two more at the C20 position; these glucose moieties are readily cleaved to produce intermediates or minor target compounds (Table 1 and Table 2) [2,6,8,9,49,50,51,52,53,54,55]. Ginsenoside bioconversion is illustrated in Figure 2.

The structures of ginsenosides used for conversion are distinguished by the type and number of sugar moieties attached to the R1, R2, and R3 sites. As noted above, PPD-type ginsenosides was classified according to positions C3 and C20, while PPT-type ginsenosides were regarded according to positions C6 and C20 [2,4,11]. Major ginsenosides are reported to have numerous health-enhancing functions, and minor ginsenosides can attack cells individually, resulting in a greater therapeutic effect [2,4,56]. Therefore, biotransformation to regenerate minor ginsenosides has been used in both research and industry. Bioconversion pathways for PPD and PPT types have been confirmed or are currently under investigation, as shown in Figure 2.

The β-glucosidase protein can be isolated from certain microorganism such as *Aspergilluls niger*, *Stereum hirsutum* JE0512, *Armillaria mellea* mycelia, and yeast; however, the most popular and effective approach is to employ bacteria [2,6,8,15,52,57,58,59]. Beneficial microorganisms such as fermented bacterial strains in human digestive system or naturally fermented bacteria from food are favored in bioconversion research due to their broad safety profiles [32,60].

## 4. β-Glucosidases Applications in Bioconversion of Ginsenosides

**Figure 3:**
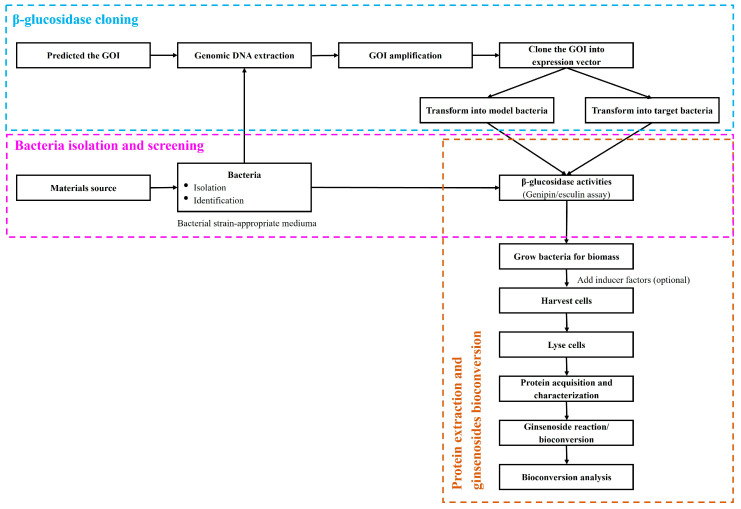
General process of ginsenoside conversion by microorganisms and recombinant enzymes. The procedure for isolating and screening bacteria and the method for cloning the recombinant enzymes are described in the pink and blue boxes, respectively. The steps involved in protein extraction and ginsenoside bioconversion are outlined in the brown box. GOI: Gene of interest.

The β-glucosidase source may be obtained from microorganisms isolated from the soil of a ginseng farm, soybean, tea, human digestive system, kimchi, and other fermented food [14,32,61,62,63,64,65,66,67,68]. While the majority of β-glucosidases capable of ginsenosides bioconversion have been identified in bacteria, similar enzyme activity has also been found fungus, and in *Aspergillus niger, Armillaria mellea*, and in particular [15,28,69,70,71]. The enzyme catalyzes the hydrolysis of glycosidic bonds to terminal non-reducing residues in β-D-glucosides and oligosaccharides, releasing glucose and transforming the major ginsenosides into the corresponding minor ginsenosides. The bioconversion of ginsenosides follows different hydrolytic pathways within a multiple-step process. The hydrolytic pathway is determined by the stereospecificity of the enzyme for C-3/C-20 or C-6/C-20 linked sugars in PPD- and PPT-type ginsenosides, respectively. These β-glycosidases yield significant quantities of minor ginsenosides because they can simultaneously hydrolyze several major ginsenosides [10,16]. A simple bioconversion process of ginsenosides by a biological method that is commonly used in the laboratory is briefly described in Figure 3. 

Ginseng plants have a long lifespan, ranging from four to six years, and the quality of their phytochemical compounds is low. Therefore, using ginseng as the primary material for large-scale industrial production is time-consuming and costly. Alternately, cost-effective sources of phytochemical compounds with similar structures and high biomass have been studied. For example, gypenosides isolated from plants, such as gypXVII compounds from *Gynostemma pentaphyllum*, are widely used for gyp LXXV or gin F2-mediated formation of CK, a well-known ginsenoside compound, exhibiting high medicinal value and various applications [31,40,56,61,63,67,72,73]. 

β-glucosidase not only induces bioconversion of ginsenosides, but has also been studied in the interaction between ginsenosides and protein domains that recognize the specific functions in living organisms such as structural or metabolic functions. Specifically, *BaBgl3B* isolated from *Bifidobacterium adolescentis* ATCC15703 acts as a biocatalytic tool for ginsenoside transformation and for the preparation of active glycosides and aglycones [31,74]. 

Several approaches to predict and identify bioconversion participants are required in order to increase applicability of the method. Such processes often involve morphological observation and screening based on the activity of a specific substrate group [44,52,58]. For example, in the genipin assay, the product of β-glucosidase hydrolysis of geniposides can react with amino acids to form a stable blue-colored compound [50]. In addition, in the esculin assay, esculin is hydrolyzed by β-glucosidase and becomes esculetin, which produces a black color in the presence of ferric ions and may act as an indicator of β-glucosidase activity. The process can be explaining line: esculin is hydrolysis by β-glucosidase and becomes the esculetin. This substrate has a black color with ferric ions [65,74,75]. Otherwise, several molecular biology tools and techniques, such as BLAST, Alignment, Illumina MiSeq sequencing, online web servers, kits, software, etc., have been used as screening methods to predict the genes responsible for high expression of target compounds. Subsequently, the dominant candidate for specificity was predicted, and their activity was confirmed by additional experiments [52,61].

## 5. Bioconversion of Ginsenosides by β-Glucosidase Enzymes Obtained from Microorganism

Bacteria, fungi, and yeast have been identified in the bioconversion of ginsenosides (Table 1) [8,20,66,67,76,77]. The isolation and purification of β-glucosidase are time-consuming and expensive processes. Therefore, whole-cell protein preparations are frequently used in the bioconversion of ginsenosides, despite the low enzymatic activity within the preparation and the presence of numerous other undefined factors. The β-glucosidase activity of a microorganism can be examined via ginsenoside conversion and minor ginsenoside synthesis. In this case, the bacteria were cultivated under artificial conditions using ginsenosides as a carbon source.

The activity of β-glucosidase can be manipulated during fermentation process by adjusting ginsenoside types, reaction concentration of enzyme or substrate, ion, pH, or temperature. Multiple studies have demonstrated that the optimal reaction conditions depend on the bacterial strains used and the rate of microbial growth that influence the reaction’s activity [5,9,10,14]. After induction of microbial biotransformation, the functionalities of fermented ginsenosides are assessed. Antioxidant activity is common among compounds extracted from medicinal plants. Compared to non-fermented ginseng, fermented ginseng shows greater hydroxyl radical scavenging and antioxidant activity. Minor ginsenosides derived from Rb1 or Rc, such as Rd, inhibit lipid oxidation and suppress the antioxidant defense system in various in vitro assays [2]. Moreover, increased total phenol or polyphenol content and increased antioxidant activity and biosynthesis in plants have been reported in fermented ginseng leaf and flower buds [2,78].

Anti-cancer activity, in particular, is of great interest for human applications. Anticancer effects of *P. ginseng* minor (but not major) ginsenoside have been demonstrated in vitro, in vivo, ex vitro, and ex vivo in both animal and human cancer cell lines [2,29,40,46,68]. Other biological activities, such as anti-inflammatory, anti-hyperglycemic, anti-diabetic, anti-aging, anti-carcinogenic, anti-fatigue, anti-pyretic regulatory effects on immunomodulation, central nervous, and cardiovascular system protection, boosting physical vitality and the promotion of DNA, RNA, and protein synthesis, also are superior in fermented compared to fresh ginseng [2,3,4,5,8,9]. 

Kimchi is a traditional Korean fermented food. *Lactobacillus* and *Leuconostoc* genera are the predominant microorganisms required for Kimchi’s fermentation. These bacteria hinder foodborne pathogen growth, viral activity, and the effects of lipid profiles but provide neuroprotective, antifungal, and antipoetic properties [14]. *Lactobacillus* genera have been demonstrated to produce Rg3 and Rg5 from Rb1, Rg1, and other organic acids [14,79]. In addition, the *Dekkera anomala* YAE-1 strain isolated from Mongolian fermented mare’s milk can produce β-glucosidase to convert Rb1 into Rd [59].

In addition to using ginsenosides as the primary source material, β-glucosidase can also be used to synthesize a few minor ginsenoside structures, although the mechanism is unknown. For example, after transformation using bacterial β-glucosidase in lactic acid bacteria from kimchi, two glucose molecules at C20 of Rb1 produce Rg3 and may contribute to the production of Rb3 and Rg5 from organic acids [14]. Otherwise, inoculation of ginseng extracts with *Stereum hirsutum* JE0512 increased the production of CK during solid-state fermentation using corn bran or cellulase from *Aspergillus niger* as the substrate [50].

Although human intestinal bacteria and edible food bacteria have tremendous potential for use in the food industry, their bioconversion requires an expensive medium for selection and has low yield and low productivity. In addition, neither the enzymes involved in the transformation nor the byproducts of the process are known [2,4,10,14,60].

**Table 1:** Bioconversion of ginsenosides by whole-cell protein extraction from microorganisms.

No.	Microorganism	Strain	Source	Pathway	Ref.
1	*Aspergillus niger*	KCCM 11239		Rb1→Rd→F2→CK Rb1→Rd→Rg3 Rb2→Rd CO→F2 CY→CK Rc→Rd Mc1→F2 Mc→CK	[28,69,70]
2	*Aspergillus niger*	XD101	Soil	Rb1→CK	[71]
3	*Armillaria mellea*	KACC 50013	Mushroom mycelia	G-Rc→C-Mc1→C-Mc G-Rc→G-Rd→G-F2→CK	[15]
4	*Chryseobacterium panacisoli* sp.	Gsoil 183^T^	Soil	Rb1→F2	[80]
5	*Chryseobacterium ginsengiterrae* sp.	DCY68^T^		Rb1→F2	[81]
6	*Chryseobacterium yeoncheonense* sp. nov.	DCY67T	Soil	Rb1→F2→CK	[82]
7	*Dekkera anomala YAE-1*	YAE-1	Mongolian Fermented Milk	Rb1→Rd	[59]
8	*Flavobacterium panaciterrae*	DCY69T	Soil	Rb1→Rd/F2	[83]
9	*Fomitella fraxinea*	KACC 42289	Korean Agricultural Culture Collection	Rb1→Rd→F2→CK Rc→C-Mc1→C-Mc→CK Rb2→CO→CY	[78]
10	*Intrasporangium* sp.	GS603	Soil	Rb1→F2 Rb1→GypXVII	[61]
11	*Lactobacillus mesenteroides*, *Pediococcus pentosaceus*	WiKim19 WiKim20	Kimchi	Rb1→Rg3	[14]
12	*Lactobacillus rossiae*	DC05	Kimchi	Rb1→CK Re→Rg2	[64]
13	*Lentilactobacillus buchneri*	URN103L	Korean fermented foods	Rb1→Rd→F2→CK Rb1→Rd→Rg3	[84]
14	*Leuconostoc citreum*	LH1	Kimchi	Rb1→Rd→F2→CK	[62]
15	*Microbacterium* sp.	GS514	Soil	Rb1→Rd→Rg3	[85]
16	*Paecilomyces Bainier* sp.	229	Soil	Rb1→Rd→Rg3	[26]
17	*Paenibacillus puernese*	DCY97T	Pu’er tea	Rb1→CK	[86]
18	*Penicillium decumbens*			Rb1→GypXVII→F2→CK Rd→F2→CK Rg3→Rh2	[40]
19	*Penicillium sclerotiorum*		Soil	Rg1→F1	[29]
20	*Weissella hellenica*	DC06	Fermented food (radish and cabbage)	Rb1→Rg3 Rb1→Rd→F2→CK	[65] [87]

## 6. Bioconversion of Ginsenoside by Recombinant β-Glucosidases 

While whole-cell preparations for ginsenosides biotransformation are fairly simple to prepare and use, it is difficult to regulate off-target processes and perform glycosides hydrolysis for selective enrichment of ginsenosides. It is also difficult for the food and cosmetics industries achieve a large-scale safe approach due to challenges such as scarcity of microorganisms certified to be generally recognized as safe (GRAS), the difficulty of scaling up fermentation, the slow reaction rate, and the presence of novel end products [2,9,10]. These restrictions can be overcome by using purified recombinant β-glucosidase, which has been shown to be extremely effective and has a short reaction time to high yields. Studies using β-glucosidase recombinant enzymes have shown successful conversion of ginsenosides almost reported in GH1 and GH3 family (Table 2). Differences in amino acid sequence, structure, and interactions are among the many factors that contribute to the specificity of enzymes with substrates [5,10,24,28]. Furthermore, the specificity of β-glucosidase with regard to ginsenoside sugar moiety enables directed ginsenosides bioconversion to produce desired minor ginsenosides. This property enables hydrolytic enzymes to catalyze ginsenoside bioconversion by cleaving the glycosyl group of major ginsenosides at the exact, specified linkage point, resulting in the anticipated minor ginsenosides [10,23,74,88]. 

Nonetheless, the lack of acceptable hots for recombinant expression of β-glucosidase has hampered the adoption of food-grade preparations. The majority of studies of recombinant β-glucosidase gene expression and bioconversion of ginsenosides continues to employ the *E. coli* system as a host, which is a significant barrier to the continued implementation of recombinant enzymes in high-quality food products. New systems to achieve large-scale conversion are needed.

Several landmark studies of β-glucosidase expression in GRAS organism have hinted at its potential for enhancing the nutritional content and quality of food, pharmaceuticals, and functional foods [46,57,61,65,80]. However, there are limitations that must be overcome for the large-scale use of β-glucosidase in these ways. There are extreme circumstances, such as pH, temperature, and concentrations of enzyme and substrate, and the difficulty of isolation and purification recombinant enzyme applications [33,36,38,42,44,48,63,68,72,73,88,89]. Consequently, determination of optimal reaction condition for bioconversion to achieve the required level of production required time and cost. Regardless, recombinant enzyme models are highly applicable in the pharmaceutical, functional food, and cosmetic industries [49]. The recombinant enzyme was summarized in Table 2 with a more specific and well-characterized biotransformation pathways.

**Table 2:** Biotransformation of ginsenosides using β-glucosidase recombinant enzymes.

No.	Microorganism	Gene	Gene/Protein ID	Family	Bioconversion Pathway	Ref.
1	*Actinosynnema mirum* KACC 20028T	bglAm	YP_003100744	GH3	Rb1→GypXVII→GypLXXV/F2 Rd→F2→Rh2(S) Re→Rh2(S) Rg1→Rh1(S)	[72]
2	*Bifidobacterium adolescentis ATCC15703*	BaBgl1A BaBgl3A			Rb1→Gyp XVII Rd→F2 Rb1→Gyp XVII and F2	[31]
3	*Bifidobacterium breve* ATCC 15700	BbBgl	CP006715.1 (1366947 to 1368227)	GH1	Rd→F2→CK	[48]
4	*Caldicellulosiruptor bescii* DSM 6725	β-glucosidase	ACM59590	GH1	Rb1/Rb2/Rc→Rd→F2→CK	[52]
5	*Flavobacterium chilense*	BglFc	SHL96941.1	GH3	Rb1→Rd→Rg3(S)→Rh2(S) GypXVII →GypLXXV→CK F2→CK Rb2 →CO→CY Rb3→C-Mx1→C-Mx Rc→C-Mc1→C-Mc Re→Rg2(S) Rg1→Rh1(S)	[44]
6	*Flavobacterium johnsoniae*	bglF3	ABQ03809	GH3	Rb1→Rd GypXVII→F2	[63]
7	*Lactobacillus brevis*	Bgy1	BAN07577	GH3	GypXVII→GypLXXV→CK	[56]
8	*Lactobacillus brevis*	bgy2	BAN05876	GH3	Rb1→Rd F2→CK	[42]
9	*Lactobacillus insenosidimutans* EMML 3041T	GST-BglL. gin-952	WP_053084464	GH3	Rb1→Rd→Rg3(S)	[68]
10	*Microbacterium esteraromaticum*	Bgp1	AEX88466.1	GH3	Re→Rg2; Rg1→Rh1 Rb1→Rd→20(S)-Rg3	[7,88,90]
11	*Microbacterium esteraromaticum* GS514	Bgp2	EU036992.1	GH2	Rb2→20(S)-Rg3	[91]
12	*Microbacterium esteraromaticum*	bgp3	JN 603821	GH3	Rb1→Rd→CK	[74]
13	*Microbacterium* sp. Gsoil 167	BglG167b	~WP_018187396	GH3	GypXVII→GypLXXV	[66]
14	*Microbacterium testaceum* ATCC 15829	MT619			Re→F1 Rb1→CK	[49]
15	*Niabella ginsenosidivorans*	BglNg-767	CP015772	GH3	Rb1→GypXVII→F2 Rd→F2 Rb2→CO Rb3→C-Mx1 Rc→C-Mc1	[73]
16	*Paenibacillus mucilaginosus* 3870T	BglPm	AEI42200	GH1	Rb1/Rd→F2	[92]
17	*Pseudonocardia* sp. Gsoil 1536	BglPC28	JX960416	GH3	Re→Rg2(S) Rg1→Rh1(S) Rb1/Rd/Rb3→Rg3(S)	[41]
18	*Thermotoga petrophila* DSM 13995	Tpexyl3 Tpebgl3	CP000702.1	GH3	Ginsenoside extract→20(S)-Rg3	[36,89]
19	*Thermotoga thermarum* DSM 5069T	Tt-BGL	YP 004660190.1	GH1	Rb1→Rd	[93]

## 7. Other Methods for Ginsenoside Bioconversion

As noted above, ginsenosides may be converted not only by biological methods but also by physical and chemical approaches. Many mechanisms are involved in the physical transformation of saponin composition, notably of the sugar moiety. In addition, several physical methods can successfully transform ginsenosides, namely heating, steaming, air-drying, sulfur fumigation, high hydrostatic pressure (HHP), and microwave treatment. However, with the exception of steaming, these processes have not yet been applied commercially [12,29,68,84,94]. Regarding chemical methods, acidic and alkaline hydrolysis at high temperatures, high pressure, and high pH has been used to the cleave or degrade major ginsenosides into minor ginsenosides for increased biological and pharmacological activity. Nonetheless, it is challenging to control the side reactions and implement the hydrolysis of glycosylation for selective ginsenoside enrichment [8,12,95].

In certain instances, physical and chemical methods are combined with enzyme activity to significantly boost bioconversion activities [5,84]. However, these methods are generally insufficiently selective, and involve unwanted side reactions, such as epimerization, hydration, and hydroxylation, and environmental pollutants. In addition to conversion, product optimization and purification are also essential. These highly productive and cost-effective processes rely heavily on physical and chemical techniques and are among the most prevalent approaches in biological resource conversion and utilization [53].

## 8. Potential Application of β-Glucosidases and Converted Ginsenosides

The process of β-glucosidase-mediated generation of minor ginsenosides from major ginsenosides illustrates the importance of ginsenoside metabolism in research and therapeutic applications [9,16,77,96]. The identification and isolation of bacterial endophytes from *P. ginseng* and *P. notoginseng* have provided novel sources of both major and minor ginsenosides, with the latter based on β-glucosidase activity [9]. The major goals in practical application are mass production and nanoparticle preparation of minor ginsenosides with large pharmacological effects [96,97]. Novel forms of minor ginsenosides may have enormous promise as therapeutic agents [96]. Functionalization or loading of minor ginsenosides onto biocompatible polymers and nanoformulations improved the solubilization and absorption of ginsenosides for clinical applications such as cancer therapy [97]. 

Research addressing the possible applications of ginsenosides in dairy products offered to Korean customers represents another potential application [16]. Lactic acid bacteria in dairy products can convert major ginsenosides into minor ginsenosides, and both panax ginseng and red ginseng can impact the physicochemical, sensory, ginsenoside content, and functional qualities of milk, yogurt, and fortified cheese [16]. Moreover, gram-scale ginseng extracts were employed as substrates for β-glucosidase activity in a GRAS strain that has been extensively exploited in the commercial production of food additives. In the beverage industry, β-glucosidase assists in the separation of aromatic compounds from the precursors of glucosides found in fruit juices and musts for wine making, as well as in the flavoring of tea and fruit juice [98]. These methods offer a low-risk, simple, and productive process that may be exploited to great effect in the development of recombinant enzymes for the food industry [16,49,98]. 

The utility of β-glucosidase has not only been documented in the food industry, but has also been documented in a wide variety of agricultural and industrial applications. For example, the geniposide present in gardenia fruits can be hydrolyzed by β-glucosidase to make genipin. Genipin combines with amino acids to produce gardenia blue, a naturally occurring blue pigment that is utilized extensively in the textile and pharmaceutical industries [99]. 

Interestingly, β-glucosidases may also be a cost-effective option to produce biofuels through cellulose saccharification using waste lignocellulosic residues, reducing the accumulation of this material in the natural environment [98,100]. Examples are application of the β-glucosidase produced by *Moniliophthora perniciosa* can or *Anoxybacillus flavithermus *subsp.* yunnanensis* E13^T^ applied in the hydrolysis of sugarcane bagasse. *Aspergillus terreus* can be utilized for the hydrolysis of soybean isoflavones. The use of many thermostable and thermophilic β-glucosidases in cellulose hydrolysis is also of particular interest because the increase in temperature during the process does not impede the activity of protein, and it also favors the hydrolysis process that is carried out on the cellulose. Notably, the thermostable β-glucosidase from *Aspergillus fumigatus* Z5 removes phenolic compounds, so it can be used to degrade polyphenols in lignocellulosic biomass. The thermophilic β-glucosidase from *Thermotoga naphthophila* RUK-10 was combined with cellulase for the hydrolysis of untreated corn straw, and the conversion rate from cellulose to glucose increased by 93.5% [98,100].

Significant β-glucosidase activity has been confirmed in several bacteria genera. However, the specific metabolism and function-determining gene involved in ginsenoside biotransformation have not been studied. Nevertheless, the method for isolating protein from whole-cell bacteria is quite simple, inexpensive, and biomass is abundant. It can be utilized in the cosmetics and detergent industries, as well as for basic conversion research. These characteristics indicate the viability and ecological sustainability of microbial production of pharmaceuticals, cosmetics, and detergents (Table 1) [28,53,60,70,75,79,81,84,101]. Certain bacteria isolated from kimchi and other foods, which are confirmed to be capable of biologically converting ginsenosides through β-glucosidase activity, may be useful for the practical preparation of minor ginsenosides in a variety of industries. They may be used to improve the nutrition and quality of food, as well as industrial pharmaceuticals and functional foods [9,46,57,60,61,65,72,78,87].

The identification of the enzymes responsible for ginsenoside biosynthesis would present new avenues for efficient large-scale production of ginsenosides. Numerous studies have demonstrated the specific activity of β-glucosidase in ginsenoside biotransformation; a summary of this research is provided in Table 2. The presence of recombinant ginsenoside-transforming β-glucosidases in GRAS host trains (*Corynebacterium glutamicum*, *Saccharomyces cerevisiae*, and *Lactococus lactis*) has been widely exploited in the industrial synthesis of food additives [43,46,55] and may be exploited to scale up production of high-value minor ginsenosides for use in the functional foods, cosmetics, and pharmaceutical industries. This will replace the *E. coli* expression system, which has been shown to be the main disadvantage for animal and human applications [43,53,55]. Furthermore, combined approaches to promote efficient production of specified minor ginsenosides are of considerable benefit in various industries, particularly sustainable long-term production of biofuels [30,53]. Moreover, in order to fulfill the ever-increasing demand, efforts to increase the activity of β-glucosidases are ongoing; possible alternatives to increase the production of valuable ginsenosides and products of biotransformation include the use of synthetic biology and metabolic engineering. The eco-friendly characteristics of β-glucosidase-mediated reactions should be acknowledged as a significant advance in both manufacturing processes and industrial applications.

## 9. Conclusions

Ginsenosides are specialized saponins found only in *Panax* species with biological and pharmacological activity, engendering their application in the food and food additive industries, cosmetic industries, pharmaceutical industries, and in healthcare. In this article, we provided a comprehensive overview of the literature addressing ginsenosides and their biotransformation, with special emphasis on the role of microbial or recombinant β-glucosidases. The β-glucosidase enzymes are present in all living species and play many roles in biotransformation processes, and they are present in all living species. Increased production of minor ginsenosides, which may boost their nutritional content and activity, improving human health and quality of life, may be achieved through the use of recombinant β-glucosidases enzyme derived from a GRAS host strain. Together, these advances have led to a cost-effective and efficient industrial platform to manufacture ginsenosides for future research and increase their therapeutic applications.

## Data Availability

Data Availability Statements are available in section “MDPI Research Data Policies” at https://www.mdpi.com/ethics.
